# Food Hydrocolloids: Structure, Properties, and Applications

**DOI:** 10.3390/foods13071077

**Published:** 2024-04-01

**Authors:** Yanlei Gao, Ru Liu, Hongshan Liang

**Affiliations:** 1 College of Food Science and Technology, Huazhong Agricultural University, Wuhan 430070, China; gyl19970925@163.com (Y.G.); lianghongshan@mail.hzau.edu.cn (H.L.); 2 Engineering Research Center of Green Development for Conventional Aquatic Biological Industry in the Yangtze River Economic Belt, Ministry of Education, National R&D Branch Center for Conventional Freshwater Fish Processing (Wuhan), Wuhan 430070, China

## 1. Introduction

Hydrocolloids are extensively used in the food industry for various functions, including gelling, thickening, stabilizing foams, emulsions, and dispersions, as well as facilitating the controlled release of flavor. In fact, the routine application of hydrocolloids mainly relies on their ability to regulate the rheology and texture of food systems, although they typically exist in small amounts at concentrations below 1%. These properties are closely related to the hydrocolloid structure and interaction between different molecules. The use and design of colloidal particles could not only improve the physicochemical properties of foods but significantly affect the nutritional value of the whole system. Likewise, hydrophilic colloids are widely used in the delivery of bioactive substances, fat replacement, salt reduction, glycemic reduction, and inhibition of safety hazards in food. Moreover, they enhance satiety, regulate obesity, modulate the glycemic index, and immunomodulate and maintain colon health. Thus, the structure, properties, and applications of hydrophilic colloids are of great significance in ultimately affecting the quality and health attributes of foods.

## 2. Sources and Classification of Hydrocolloids

Hydrocolloids are a series of hydrophilic macromolecular substances that mainly originate from microbial metabolism (like xanthan gum), plant seeds (like guar gum and acacia bean limbs), tree secretion (like gum arabic), fruit rinds (like pectin), animal colloids (like gelatin), algae (like carrageenan and alginate), cellulose derivatives, and modified starch [1]. They can form slippery, viscous, or jelly-like solutions after absorbing water [2]. Hydrocolloids are commonly used in food for their unique functional, nutritional, and safety properties.

## 3. Functional Properties

### 3.1. Thickening Properties

In food systems, the high affinity of hydrocolloids for water molecules significantly changes the rheological properties of food [3]. Rheological properties are defined as mechanical properties that cause food deformation and flow in the presence of stress. Thickening reduces the fluidity of food, essentially enhancing the interaction between macromolecules and solvents [4]. Hydrophilic colloids with different molecular structures and concentrations exhibit different viscosities [5]. Specifically, macromolecules are free to move in solution at low hydrocolloid concentrations, while they become entangled above overlapping concentrations [6]. The longer the molecular chain of hydrocolloids, the higher the frequency of intermolecular collision and friction, and the more viscous colloidal solutions could be formed [7]. Recently, it was reported that flaxseed gum extracted from flaxseed shells has a high water-binding capacity, which could be used as a thickener for beverages and bread [8].

### 3.2. Gel Properties

#### 3.2.1. Animal Protein Gels

Myofibrillar proteins (MP) play an important role in the gelling of meat. The textural properties of meat products are intently linked to the three-dimensional network structure of MP [9]. Some treatments could destroy or improve their gel network structure. For example, rapid freezing and cold thawing could lead to increased droplet loss in crayfish meat, resulting in the formation of rough, irregular, and larger pore microstructures [10]. Exogenous additives (e.g., oil, proteins, starch, and hydrocolloids) or emerging technologies (e.g., ultrasound, ultrahigh pressure, electrostatic field, and electron irradiation) have been utilized to enhance the gel properties of meat products. It was reported that the addition of myofibril pre-emulsified oil enhanced the gel strength and water retention of shrimp gel [11]. Gao et al. [12] found that ultrasonic treatment could significantly enhance the gel properties of silver carp myosin with low NaCl contents. Additionally, the addition of hydrophilic colloids (carrageenan, xanthan gum, konjac gum, etc.) could also enhance the gel properties of MP. Heating can cause MP to expose active groups, leading to enhanced intermolecular interactions and consequently to the formation of the MP gel network structure [13]. Meanwhile, hydrophilic colloids undergo rearrangement within the MP gel system, filling gel pores and forming gel segments [14]. Moreover, the high hydrophilicity of colloids can reduce the exudation of random moisture caused by protein denaturation, which leads to better polymerization of the MP network [15].

#### 3.2.2. Plant Protein Gel

The hydration behavior of protein molecules during heating leads to the aggregation of molecules [16]. Throughout the process of aggregation, there exists a balance between the attraction and repulsion of molecules, resulting in the formation of a structured three-dimensional network [17]. However, most plant proteins are sensitive to environmental factors such as pH, temperature, and ionic strength, resulting in gel products with loose structures, molding difficulties, low yields, and poor water retention [18]. Recently, some scientists have concentrated on improving the gel properties of plant proteins through the addition of hydrocolloids. Hydrocolloids can cross-link plant proteins through non-covalent bonds and other interactions, play a synergistic effect in the formation of plant protein gels, and significantly improve the gel properties of food [19]. It was found that an appropriate concentration of konjac gum increased the viscoelasticity of wheat gluten, glutenin, and gliadin gels [20].

### 3.3. Stability and Emulsification

The emulsion is prone to phase separation due to gravity separation, flocculation, or agglomeration [21]. Hydrophilic colloids are conducive to the formation and stability of emulsions by reducing interfacial tension [22]. An effective emulsifier requires higher surface activity. It should quickly absorb into the oil-water interface to reduce interfacial tension and enhance short-term stabilization [23]. This tension reduction is closely related to the time scale of the emulsion preparation process. In other words, the rate of absorption of colloids on the oil-water interface should exceed the rate of droplet collisions induced by the hydrodynamic forces of laminar and turbulent flow [24]. Moreover, they could also act as a barrier against oil droplet aggregation to retard the settling of dispersed particles and further prevent the dehydration and shrinkage of gels [25]. The stabilizers with limited absorption capacity maintain the long-term stability of the emulsion by binding to the interface or changing the viscosity of the continuous phase [26]. Sutariya et al. [27] revealed that a mixture of hyaluronic acid and carrageenan significantly improved the storage stability, heating stability, and emulsification properties of milk.

## 4. Application of Food Hydrocolloids to Nutritional Security Properties

### 4.1. Low Salt, Low Fat, and Low Glycemic Index

#### 4.1.1. Salt-Reducing

The global population’s salt intake varies from 9 g/day (equivalent to 3.54 g of sodium) to 12 g/day (equivalent to 4.72 g of sodium) [28], surpassing the World Health Organization’s suggested daily limit of 5 g/day (equal to 2 g of sodium) [29]. Excessive sodium intake increases the risk of hypertension. However, sodium is not only a key factor in imparting saltiness but also acts as a processing aid, controlling water activity, and improving the texture of foods [30]. In the food system, the volume of the hydrophobic phase and the distribution area of salt have a significant impact on the perception of saltiness [31]. Therefore, the perception of saltiness could be enhanced by adjusting the matrix structure and composition, controlling processing performance [32], boosting interface instability [33], and increasing the synergistic use of multiple flavors [34]. Food colloids are a mixture dispersion system that could effectively control the transfer and release of bioactive ingredients [35]. The use of hydrocolloids to reconstruct food structure could reduce the release of salt, increase saltiness perception, and ultimately achieve the goal of “reducing salt without reducing saltiness” in food [32]. Li et al. [36] found that large amounts of gum arabic decreased the salt content of yogurt drinks and mayonnaise by approximately 30% without reducing the perception of saltiness. They speculated that gum arabic, as a mucus-permeable polysaccharide, could minimize the hindrance of mucin to facilitate sodium ions into taste receptors in the oral cavity, thereby enhancing saltiness perception. For breads, cheeses, and sausages, protein/polysaccharide aggregates act as carriers to transport sodium, which reduces the total sodium content through the non-uniform distribution of salt in the food. According to reports, coarse-grained salt [37] and encapsulated salt [38] could significantly reduce total salt content without changing saltiness perception or the overall acceptability of bread by forming alternating salt layers.

#### 4.1.2. Glycemic-Lowering Properties

In the development process of low-glycemic foods, the glycemic index (GI) is an important characterization parameter. Foods with a GI below 55 are typically referred to as low-GI foods [39]. There are two main technical routes for the development of low-GI foods: (1) changing the content of carbohydrates in food; (2) controlling the rate of carbohydrate breakdown in food. The addition of hydrocolloids could improve the delayed retrogradation of starch. The intake of high-content amylose is beneficial for suppressing glycemic levels in foods [40]. It was found that konjac glucomannan inhibited the short-term and long-term retrogradation of amylose. This is mainly attributed to the high hydrophilicity of konjac glucomannan, which prevented water molecules from participating in the rearrangement of amylose [41]. Furthermore, the decomposition rate of carbohydrates could be accelerated with the aid of hydrophilic colloids or process technology. Zeng et al. [42] found that the addition of yeast β-glucan and proteins significantly inhibited the gelatinization and retrogradation of starch in whole-wheat bread, resulting in a lower glycemic index. Similarly, Xiong et al. [43] reported that microwave-cooked starches showed more short-range double helices, long-range microcrystals, and nanoscale units than conventionally cooked starches. Notably, little is known about the role of hydrocolloids in the human gastrointestinal tract, leading to uncertainty about the exact mechanism of their prevention of diabetes.

#### 4.1.3. Fat-Lowering Properties

Fats play an important role in providing the texture and flavor of food. It exists in the form of colloidal particles (fat droplets) in milk or cream, which could increase the viscosity and perception of cream flavor [44]. Fat could also improve the texture of solid foods such as bread, cake, and cookies by forming a 3D network of fat crystals [45]. However, excessive intake of fats (especially saturated and trans fats) increases the risk of a series of chronic diseases. The World Health Organization recommends limiting the intake of total fat to not exceed 30% and saturated fat to below 10% of total energy intake [46]. Although directly reducing the additive amount of fat in food could decrease fat intake, it would seriously degrade the flavor and texture of food [47]. At present, fat reduction technology mainly relies on food formula and processing conditions based on fat replacements [48]. Common fat replacements are proteins and carbohydrates. Proteins are often processed into microgel particles with a diameter of 0.1 to 2.0 μm so that they do not feel grainy and melt quickly in the mouth, which significantly reduces fat contents without affecting the overall texture and taste of the food [49]. In addition, polysaccharide colloids offer a new method. They simulate fat properties in the form of colloidal particles, high-internal-phase emulsions, oleogels, and emulsion gels by forming oil-free colloidal systems and structured oil systems [50]. They could also interact with other polysaccharides, proteins, polyphenols, lipids, and other components to enhance the physicochemical properties of polysaccharide colloids, which in turn improves the quality of fat-reduced foods. It was reported that the ethanol-regulated konjac emulsion after freeze-thaw has similar mechanical and physicochemical properties to pork back fat [51]. Using a blend of soy protein hydrolysate and xanthan gum to replace 50% of the fat in ice cream results in a product that closely resembles full-fat ice cream in terms of appearance, flavor, and texture [52].

### 4.2. Bioactive Material Delivery

Bioactive substances have a variety of advantages, such as antioxidant, anti-inflammatory, and antibacterial properties [53]. Their incorporation as dietary supplements could enhance the functional properties of foods [54]. However, many hydrophilic bioactive substances (water-soluble pigments, spices, nutraceuticals, and medicines) and lipophilic bioactive substances (seaweed oil, curcumin, and vitamin A) have poor physicochemical stability or strong peculiar odors [55]. The hydrophilic bioactive ingredient could be encapsulated in a gel system assembled from proteins and polysaccharides. Meanwhile, hydrophilic colloids employing covalent coupling, or nanocarrier structures, are widely used to load bioactive substances to prevent oxidation and increase their dispersibility and bioavailability [56]. Liu et al. [57] discovered that combining sodium alginate and whey protein to create a composite emulsion gel greatly enhanced the photostability, gastrointestinal stability, and anti-inflammatory properties of lycopene. Liu et al. [57] found that a composite emulsion gel formed by coupling sodium alginate and whey protein significantly improved the photostability, gastrointestinal stability, and anti-inflammatory activity of lycopene. Lipophilic bioactive ingredients could be encapsulated in oil-water emulsions composed of protein-based conjugates, which could effectively protect and transport probiotics [58]. Furthermore, the selection of a suitable carrier depends on the characteristics of the delivery material. For instance, hydrophilic gel microspheres could be used to load hydrophilic small molecules (e.g., active peptides) [59].

### 4.3. Food Intelligent Packaging

Food that comes into contact with the environment during packaging or processing causes microbial contamination, which reduces the shelf life and safety of food. Some researchers have added antimicrobial agents to macromolecule films for food packaging applications [60,61]. However, antimicrobial packaging cannot stop food spoilage, which may occur at any time, posing a challenge for consumers to promptly assess food quality. Therefore, intelligent packaging has become a research hotspot. It could perceive and monitor temperature, oxygen, pH, microorganisms, and certain chemicals (such as volatile alkaline nitrogen) in food [62]. By observing the state of packaging materials, consumers can avoid wasting food to some extent. Types of intelligent identification food packaging were film, label, and barcode [63]. They consist of sensor materials and macromolecule-based materials (protein or polysaccharide) forming a highly compatible mixture matrix under certain processing conditions [64]. Protein-based materials include collagen, zein, and soybean isolate proteins. Polysaccharide-based materials comprise gellan gum, starch, chitosan, sodium alginate, and branched-chain starch [65]. They are widely used as biocomposites for their excellent biodegradability, biocompatibility, and security [66]. Musso et al. [67] prepared pH-sensitive smart food packaging materials by mixing anthocyanins (sensors) with film-forming gelatin solutions. An H_2_S sensor was fabricated using silver nanoparticles coated with gellan gum, leveraging Ag’s high binding affinity for H_2_S. This sensor demonstrated a transition in color from yellow to colorless up-on detection of H_2_S levels generated from chicken breast and silver carp [68].

### 4.4. Safety Hazard Factor Suppression

Deep-fried or roasted foods have a crispy texture and unique taste, which are deeply loved by young people. However, these foods have high oil content and lipid oxidation, resulting in the production of harmful substances such as advanced glycation end products (AGEs) and acrylamide [69]. Excessive intake of these substances would induce diseases such as obesity, hypertension, and hyperlipidemia [70]. Currently, many studies have focused on modifying food structure through the addition of functional substances to prevent moisture loss and oil absorption [71,72]. Hydrophilic colloids could form a certain three-dimensional network structure in an aqueous solution through hydrogen bonding, van der Waals forces, or ionic interaction. Water molecules would be bound within the network structure and lose their mobility while inhibiting oil absorption [73]. Sun et al. [74] reported that the addition of konjac glucomannan significantly reduced the AGEs and acrylamide contents of fried fish nuggets.

## 5. The Development Prospects of Hydrophilic Colloids in Food

The addition of hydrocolloids not only improves the functional and sensory characteristics of food but also provides beneficial nutritional health benefits. It is well known that structure determines functional properties. Currently, designing hydrophilic colloidal structures to regulate the physicochemical properties of food has been a hot and difficult topic. This is a very promising field for the development of smart biomaterials. In addition, hydrocolloids have a variety of chemical functional groups and biological properties that allow for the effective controlled release of bioactive substances, making it necessary to understand their drug delivery applications. Meanwhile, nanotechnology offers additional possibilities for controlled and target-specific drug delivery. This provides new ideas for the design and development of carrier structures for functional food nutrients and opens up new avenues for exploring the regulatory mechanisms of hydrophilic colloids on food quality.

In this book, we present the functional properties of hydrocolloids and their applications as thickeners, gelling agents, stabilizers, and emulsifiers in different foods. In addition, nutritional safety properties of hydrocolloids in food were introduced in terms of low-fat (taking ice cream as an example), low-salt (taking yogurt drinks, mayonnaise, bread, cheeses, and sausages as examples), low-glycemic index (taking starch-based foods as an example), bioactive substance delivery (taking lycopene, probiotics, and active peptides as examples), food intelligent packaging (taking chicken breasts as an example), and food safety hazard factor inhibition (taking fried fish nuggets as an example). Finally, based on the compositions and unique properties of hydrophilic colloids, the following potential directions for future in-depth research were discussed: (1) The physicochemical properties of food might be effectively regulated by modifying the hydrophilic colloid structure to alter its interaction with food components; and (2) hydrophilic colloids might effectively deliver targeted and specific drugs while transporting nutrients in food matrices.

This book is designed for food science and technology students. We sincerely appreciate the dedication and outstanding contributions of the authors. We trust that readers will find value in this book and encourage constructive feedback, including the identification of any inevitable typographical errors.
